# Characterization and Antimicrobial Activity of Volatile Constituents from Fresh Fruits of *Alchornea cordifolia* and *Canthium subcordatum*

**DOI:** 10.3390/medicines3010001

**Published:** 2015-12-29

**Authors:** Emmanuel E. Essien, Jennifer Schmidt Newby, Tameka M. Walker, William N. Setzer, Olusegun Ekundayo

**Affiliations:** 1 Department of Chemistry, University of Uyo, Akwa Ibom State 520101, Nigeria; 2 Department of Chemistry, University of Alabama in Huntsville, Huntsville, AL 35899, USA; jennifer.newby315@gmail.com (J.S.N.); Ivygrad08@yahoo.com (T.M.W.); wsetzer@chemistry.uah.edu (W.N.S.); 3 Department of Chemistry, University of Ibadan, Ibadan 200284, Nigeria; oekundayo@yahoo.com

**Keywords:** *Alchornea cordifolia*, *Canthium subcordatum*, essential oil composition, antibacterial, antifungal

## Abstract

Bacterial resistance has been increasingly reported worldwide and is one of the major causes of failure in the treatment of infectious diseases. Natural-based products, including plant secondary metabolites (phytochemicals), can be exploited to ameliorate the problem of microbial resistance. The fruit essential oils of *Alchornea cordifolia* and *Canthium subcordatum* were obtained by hydrodistillation and analyzed by gas chromatography-mass spectrometry (GC-MS). The essential oils were subjected to *in vitro* antibacterial, antifungal and cytotoxic activity screening. Thirty-eight compounds comprising 97.7% of *A. cordifolia* oil and forty-six constituents representing 98.2% of *C. subcordatum* oil were identified. The major components in *A. cordifolia* oil were methyl salicylate (25.3%), citronellol (21.4%), α-phellandrene (7.4%), terpinolene (5.7%) and 1,8-cineole (5.5%). Benzaldehyde (28.0%), β-caryophyllene (15.5%), (*E*,*E*)-α-farnesene (5.3%) and methyl salicylate (4.5%) were the quantitatively significant constituents in *C. subcordatum* fruit essential oil. *A. cordifolia* essential oil demonstrated potent *in vitro* antibacterial activity against *Staphylococcus aureus* (MIC = 78 μg/mL) and marginal antifungal activity against *Aspergillus niger* (MIC = 156 μg/mL). *C. subcordatum* showed antibacterial activity against *Bacillus cereus* and *S. aureus* (MIC = 156 μg/mL) and notable antifungal activity against *A. niger* (MIC = 39 μg/mL). However, no appreciable cytotoxic effects on human breast carcinoma cells (Hs 578T) and human prostate carcinoma cells (PC-3) were observed for either essential oil. The antimicrobial activities of *A. cordifolia* and *C. subcordatum* fruit essential oils are a function of their distinct chemical profiles; their volatiles and biological activities are reported for the first time.

## 1. Introduction

Antimicrobial resistance is one of the most serious public health threats that results mostly from the selective pressure exerted by antibiotic use and abuse [1,2]. During the last few decades, rapid evolution and spread of resistance among clinically important bacterial species have been observed. Due to this increasing resistance, many antimicrobial agents are losing their efficacy [3,4,5]. Consequently, the therapeutic options for the treatment of infections have become limited or even unavailable. According to the World Health Organization (WHO), infectious diseases are the second cause of death around the world [6]. Therefore, it is necessary to search and develop new alternative compounds to ameliorate the problem of microbial resistance.

*Alchornea cordifolia* (Schumach. and Thonn.) Müll. Arg. (Euphorbiaceae) is a shrub found along the coastal regions of West Africa. It has multipurpose utilization as fodder, food and medicine. The leaves, roots and stem bark extracts are used extensively in traditional medicine in the preparation of drugs for urinary, respiratory and gastro intestinal disorders [7]. A slurry from the fruits is administered for asthma and cough. The leaves are used internally for the management of gastrointestinal, respiratory and urinary tract infections and externally for wounds. A decoction of the leaves is used as eye lotion [8]. The leaves and stem bark, when powdered, are used in the treatment of ringworms and other skin infections [9]. Hitherto, the leaf part of *A. cordifolia* has been a subject of scientific studies: anti-microbial, antioxidant and anticancer activities, *etc.* [10,11,12,13]. The gas chromatographic—mass spectral (GC-MS) characterization of the volatile oil from fresh leaves of *A. cordifolia* has been reported [14]. The aqueous extract of *A. cordifolia* has demonstrated antibacterial activity against 21 bacterial strains tested and showed the highest levels of antibacterial activity with MICs against methicillin-resistant *Staphylococcus aureus* (MRSA) in the range of 1.6–3.1 mg/mL and MBCs in the range of 6.3–12.5 mg/mL among 24 other plant species studied [15]. Phyto-constituents, such as steroids, phenolic compounds, flavonoids, flavones, tannins, xanthones and alkaloids, have been isolated from *A. cordifolia* leaf [16,17,18,19].

*Canthium subcordatum* DC. (formerly *Psydrax subcordata* DC., Rubiaceae) is a tree that grows in central and western Africa and reaches a height of more than 10 m [20]. Its roots, leaves and stem bark are used for medicinal purposes. Alcoholic extracts of the stem bark have potential antidiabetic properties [21] and the roots are used to treat malaria fever, inflammation and cardiovascular disease [22]. Recently, five new iridoid dimers were isolated from the fruits of *C. subcordatum* [23]. A number of iridoids, which include (6*S*,9*R*)-roseoside [24] and shanzhisin methyl ester gentiobioside [25] have been isolated from the stem-bark of *C. subcordatum* and their structures deduced. GC-MS analysis and anticancer activity of ethanol leaf extract of *C. parviflorum* have also been reported [26,27]. As part of an ongoing search for biologically active essential oils from the rain forest biodiversity of Nigeria, we report the antibacterial and antifungal activities of volatile constituents from the aromatic fresh fruits of *A. cordifolia* and *C. subcordatum*.

## 2. Experimental Section

### 2.1. Plant Material

The mature fresh fruits of *A. cordifolia* and *C. subcordatum* were collected in the month of July 2004, from the campus of the University of Ibadan, Nigeria. Plant samples were authenticated by F. Usang of the Herbarium Headquarters, Forest Research Institute of Nigeria (FRIN), Ibadan, Nigeria, where voucher specimens (FHI 107409 and FHI 107410, respectively) were deposited.

The fruit essential oils were obtained by hydrodistillation (4 h) of the pulverized air-dried plant samples (500 g) in an all glass Clevenger-type apparatus following the British Pharmacopoeia specifications [28]. The fruit oils were dried over sodium sulfate and kept in refrigeration (4 °C) after estimation of percentage yield.

### 2.2. Gas Chromatographic—Mass Spectral Analysis

The essential oils were subjected to GC-MS analysis on an Agilent system consisting of a model 6890 gas chromatograph, a model 5973 mass selective detector (MSD) (Agilent Technologies, Santa Clara, CA, USA), and an Agilent ChemStation data system (http://www.agilent.com/en-us/products/software-informatics/massspec-workstations/gc-msd-chemstation-software). The GC column was an HP-5ms fused silica capillary with a (5% phenyl)-methyl polysiloxane stationary phase (30 m × 0.25 μm film thickness). The carrier gas was helium with a column head pressure of 7.07 psi and flow rate of 1.0 mL/min. Inlet temperature was 200 °C and MSD detector temperature was 280 °C. The GC oven temperature program was used as follows: 40 °C initial temperature, held for 10 min; increased at 3 °C/min to 200 °C; increased 2 °C/min to 220 °C. The sample was dissolved in CH_2_Cl_2_, and 1 μL was injected using a splitless injection technique.

Identification of individual constituents of the essential oils was achieved based on their retention indices (determined with a reference to a homologous series of normal alkanes) and by comparison of their mass spectral fragmentation patterns (National Institute of Standards and Technology, NIST, database/ChemStation data system) and with the literature [29].

### 2.3. Antibacterial Screening

*A. cordifolia* and *C. subcordatum* essential oils were screened for antibacterial activity against *Bacillus cereus* (ATCC No. 14579), *Staphylococcus aureus* (ATCC No. 29213), *Pseudomonas aeruginosa* (ATCC No. 27853), and *Escherichia coli* (ATCC No. 10798). Minimum inhibitory concentrations (MICs) were determined using the micro broth dilution technique [30]. Dilutions of the samples were prepared in cation-adjusted Mueller Hinton broth (CAMHB) beginning with 50 μL of 1% *w*/*w* solutions of samples in DMSO plus 50 μL CAMHB. The sample solutions were serially diluted (1:1) in CAMHB in 96-well plates to give concentrations of 2500, 1250, 625, 313, 156, 78, 39, and 19.5 μg/mL. Organisms at a concentration of approximately 1.5 × 10^8^ colony-forming units (CFU)/mL were added to each well. Plates were incubated at 37 °C for 24 h; the minimum inhibitory concentration (MIC) was determined as the lowest concentration without turbidity. Gentamicin was used as a positive antibiotic control; DMSO was used as a negative control (50 μL plus 50 μL CAMHB, serially diluted as above).

### 2.4. Antifungal Screening

Antifungal activity was determined, as described above for bacteria (*i.e.*, serial dilution, concentrations of 2500, 1250, 625, 313, 156, 78, 39, and 19.5 μg/mL) using *Candida albicans* (ATCC No. 10231) in a yeast-nitrogen base growth medium with approximately 7.5 × 10^7^ CFU/mL. Amphotericin B was used as the positive control. An additional test for antifungal activity against *Aspergillus niger* (ATCC No. 16888) was determined as above using yeast mold (YM) broth inoculated with *A*. *niger* hyphal culture diluted to a McFarland turbidity of 1.0. Amphotericin B was the positive control.

### 2.5. Cell Culture

Human Hs578T breast ductal carcinoma cells (ATCC No. HTB-129) [31] were grown in a 3% CO_2_ environment at 37 °C in Dulbecco’s modified Eagle medium (DMEM) with 4500 mg glucose per liter of medium, supplemented with 10% fetal bovine serum, 10 μg bovine insulin, 100,000 units penicillin and 10.0 mg streptomycin per liter of medium, and buffered with 44 mM NaHCO_3_, pH 7.35.

Human PC-3 prostatic carcinoma cells (ATCC No. CRL-1435) [32] were grown in a 3% CO_2_ environment at 37 °C in RPMI-1640 medium with l-glutamine, supplemented with 10% fetal bovine serum, 100,000 units penicillin and 10.0 mg streptomycin per liter of medium and buffered with 15 mM Hepes and 23.6 mM NaHCO_3_, pH 7.30.

### 2.6. Cytotoxicity Screening

Hs 578T cells were plated into 96-well cell culture plates at 1.0 × 10^5^ cells per well and PC-3 cells at 1.9 × 10^4^ cells per well. The volume in each well was 100 μL for both cell types. After 48 h, supernatant fluid was removed by suction and replaced with 100 μL growth medium containing either 2.5 or 1.0 μL of dimethylsulfoxide (DMSO) solution of oils (1% *w*/*w* in DMSO), giving a final concentration of 250 or 100 μg/mL, respectively, for each oil. Hs 578T cells were tested with final concentrations at 250 μg/mL and PC-3 at final concentration of 100 μg/mL. Solutions were added to wells in four replicates. Medium controls and DMSO controls (25 or 10 μL DMSO/mL) were used. Tingenone (250 or 100 μg/mL) was used as a positive control [33]. After the addition of the sample, plates were incubated for 48 h at 37 °C; medium was then removed by suction, and 100 μL of fresh medium was added to each well. In order to establish percent kill rates, the Cell Titer 96^®^AQ_ueous_ Non-Radioactive Cell Proliferation assay was performed [34]. After colorimetric readings were recorded (using a Molecular Devices SpectraMAX Plus microplate reader, 490 nm, Molecular Devices, LLC, Sunnyvale, CA, USA), average absorbances, standard deviations and percent kill ratios (%kill_oil_/% kill_DMSO_) were calculated.

## 3. Results and Discussion

The relative concentrations of the volatile components in *A. cordifolia* and *C. subcordatum* fruits, according to their elution order on HP-5ms capillary column are presented in Table 1 and Table 2, respectively. The aromatic fruit essential oils were obtained in 0.17% (*w*/*w*) yield. Thirty-eight compounds comprising 97.7% of *A. cordifolia* oil and forty-six constituents representing 98.2% of *C. subcordatum* oil were identified. The fruit essential oil of *A. cordifolia* consisted of oxygenated monoterpenoids and aromatic esters (52.9% and 26.5%), monoterpene and sesquiterpene hydrocarbons (17.2% and 14.6%) and low amounts of aliphatic alcohol and aldehyde (3.7%). The major components identified in this sample include methyl salicylate (25.3%), citronellol (21.4%), α-phellandrene (7.4%), terpinolene (5.7%) and 1,8-cineole (5.5%). Other minor constituents detected in considerable quantities were *p*-cymene (3.6%), α-humulene (3.0%), β-caryophyllene (2.8%) (*E*)-β-damascenone (2.0%) and 1-octen-3-ol (2.0%). Two uncommon essential oil constituents were identified as (*Z*)-rose oxide and geosmin in *A. cordifolia* essential oil. (*Z*)-Rose-oxide (isobutenyl-4-methyl tetrahydropyran) is a perfumery ingredient and a constituent (*inter alia*) of *Pelargonium* essential oils and secretions of *Aromia moschata* [35]. This cyclic monoterpene ether, found in 0.5% concentration in rose oil, is said to be responsible for the highly volatile floral-green top note [36]. Geosmin (1,10-dimethyl-9-decalol), detected in 1.0% concentration, is reported as a microbial volatile organic compound. Geosmin is described as a powerful aromatic compound with an earthy smell and is implicated as one of the consequences of rot on grapes. It has very low odor threshold and strong odors. Microorganisms such as fungi and bacteria (*Actinomycetes*, *Streptomyces riseus* and *Streptomyces odourifer*) are reported to be present on some fruits, grapes for example, and are known for their ability to produce geosmin during metabolism [37]. According to Dionigi *et al.* [38], geosmin is derived from a sesquiterpene precursor such as farnesyl pyrophosphate. The major constituents of the fresh leaf essential oil of *A. cordifolia* reported by Okoye *et al.* [14] were eugenol (41.7%), cadinol (2.46%), linalool (30.6%), caryophyllene (1.04%) and (*E*)-α-bergamotene (4.54%). Twenty-five constituents consisting 90.3% of the composition of the leaf essential oil were identified in 0.13% *w*/*w* yield.

Benzaldehyde (28.0%), β-caryophyllene (15.5%), (*E**,**E*)-α-farnesene (5.3%) and methyl salicylate (4.5%) were the quantitatively significant constituents in *C. subcordatum* fruit essential oil. *C. subcordatum* fruit oil is comprised of 41.3% hydrocarbons and 56.9% oxygen-containing compounds. The five classes of organic compounds identified and reported are twenty-four hydrocarbons (41.3%), twelve alcohols (18.6%), three aldehydes (28.7%), four aromatic esters (5.8%), one ketone and one ether (3.8%). The fruit volatile oil is dominated by sesquiterpenoid compounds (50.8%), followed by aromatic compounds (33.8%), monoterpenoid compounds (7.3%), simple aldehydes and alcohols (6.3%). The monoterpenoid profile consisted of two monoterpene hydrocarbons (2.8%) and six oxygenated monoterpenes (4.5%). Twenty-two sesquiterpene hydrocarbons and seven oxygenated sesquiterpenes made up the sesquiterpenoid profile of the oil. Two unusual sesquiterpenoids, α-calacorene and longiborneol were identified in *C. subcordatum* fruit oil. The sesquiterpene alcohol, longiborneol is a constituent of *Juniperus*, *Pinus*, *Cupressus*, *Dacrydium* species and *Cedrus deodara*. It is reported to be a plant growth regulator (inhibits cress root growth and promotes wheat germination) [35]. A literature search has revealed no previous work on the analyses of volatile components of this plant or other *Canthium* species. The high concentration of benzaldehyde in essential oil samples has been reported by other workers. Lei *et al.* [39] showed that essential oils from fresh flowers of *Cerasus subhirtella* and *C. serrulata* contain 31.2% and 42.1% benzaldehyde, respectively as major constituents. Benzaldehyde (96.96%) was also indicated as a major component in the leaf essential oil of *Prunus myrtifolia* [40].

**Table 1:** Chemical composition of *Alchornea cordifolia* fruit essential oil.

RI ^a^	RI ^b^	Compound ^c,d^	Area %	QI ^e^ %
855	854	(*E*)-2-Hexenal	1.7	97
940	939	α-Pinene	0.5	95
982	978	1-Octen-3-ol	2.0	89
1003	1005	α-Phellandrene	7.4	94
1023	1026	*p*-Cymene	3.6	95
1031	1033	1,8-Cineole	5.5	98
1086	1088	Terpinolene	5.7	98
1111	1111	(*Z*)-Rose oxide	0.6	93
1194	1190	Methyl salicylate	25.3	95
1221	1228	Nerol	0.8	96
1229	1228	Citronellol	21.4	97
1244	1240	Neral	0.7	96
1247	-	Isogeraniol	1.6	94
1258	1255	Geraniol	1.9	91
1272	1270	Geranial	1.0	95
1375	1376	α-Copaene	0.9	99
1385	1380	(*E*)-β-Damascenone	2.0	96
1392	1391	β-Elemene	0.4	95
1405	-	Geosmin	1.0	95
1408	1409	α-Gurjunene	0.3	99
1418	1418	β-Caryophyllene	2.8	99
1428	1426	α-(*E*)-Ionone	0.2	97
1452	1454	α-Humulene	3.0	99
1459	1461	Alloaromadendrene	0.3	99
1480	1480	Germacrene D	0.4	97
1484	1485	β-Selinene	0.7	99
1495	1494	Bicyclogermacrene	0.4	93
1509	1508	(*E*,*E*)-α-Farnesene	0.3	89
1523	1524	δ-Cadinene	0.9	99
1541	1542	α-Calacorene	0.2	98
1564	1564	(*E*)-Nerolidol	0.6	87
1579	1581	Caryophyllene oxide	1.1	98
1585	1583	(*E*)-2-Hexenyl benzoate	0.9	97
1617	1619	10-*epi*-γ-Eudesmol	0.5	98
1628	1627	1-*epi*-Cubenol	0.3	90
1642	1642	Cubenol	0.2	93
1683	1683	α-Bisabolol	0.3	94
1759	1762	Benzyl benzoate	0.3	96

^a^ RI, calculated retention indices; ^b^ RI, retention index from literature; ^c^ Order of elution on HP-5ms capillary column; ^d^ Identification by comparison of the mass spectral and retention index data; ^e^ QI, “quality index”, reflects the fit comparison of experimental mass spectrum and National Institute of Standards and Technology (NIST) library spectrum.

**Table 2:** Chemical composition of *Canthium subcordatum* fruit essential oil.

RI ^a^	RI ^b^	Compound ^c,d^	Area %	QI ^e^ %
855	854	(*E*)-2-Hexenal	0.4	90
940	939	α-Pinene	1.0	96
966	961	Benzaldehyde	28.0	95
982	978	1-Octen-3-ol	4.4	92
998	993	3-Octanol	1.2	83
1025	1026	*p*-Cymene	1.8	93
1031	1033	1,8-Cineole	1.1	98
1102	1098	Linalool	2.0	94
1175	1171	Nonanol	0.3	94
1194	1190	Methyl salicylate	4.5	95
1218	1218	β-Cyclocitral	0.3	94
1234	1235	Thymol methyl ether	0.1	96
1257	1255	Geraniol	0.8	87
1350	1351	α-Cubebene	0.7	99
1376	1376	α-Copaene	2.9	99
1384	1384	β-Bourbonene	1.1	98
1391	1390	β-Cubebene	0.4	99
1393	1391	β-Elemene	0.5	97
1398	1398	Cyperene	0.2	90
1410	1409	α-Gurjunene	0.3	99
1418	1418	β-Caryophyllene	15.5	99
1429	1432	Calarene	0.5	92
1437	1436	α-Bergamotene	0.1	95
1439	1439	α-Guaiene	0.2	99
1453	1454	α-Humulene	1.2	99
1456	-	Nerylacetone	0.2	95
1460	1458	(*E*)-β-Farnesene	0.1	91
1468	1469	Drima-7,9(11)-diene	0.4	97
1478	1477	γ-Muurolene	1.1	99
1482	1480	Germacrene D	2.1	98
1486	1485	β-Selinene	1.3	99
1496	1491	Valencene	1.3	92
1501	1499	α-Muurolene	0.8	98
1511	1508	(*E*,*E*)-α-Farnesene	5.3	95
1524	1524	δ-Cadinene	2.1	99
1543	1542	α-Calacorene	0.4	91
1567	1564	(*E*)-Nerolidol	0.8	91
1572	1570	(*Z*)-3-Hexenyl benzoate	0.5	98
1583	1581	Caryophyllene oxide	3.5	90
1586	1583	(*E*)-2-Hexenyl benzoate	0.4	90
1596	1592	Longiborneol	2.3	98
1629	1627	1-*epi*-Cubenol	1.3	87
1643	1642	Cubenol	0.9	95
1648	1645	Torreyol (=α-Murrolol)	1.9	93
1656	1653	α-Cadinol	1.6	83
1760	1762	Benzyl benzoate	0.4	98

^a^ RI, calculated retention indices; ^b^ RI, retention index from literature; ^c^ Order of elution on HP-5ms capillary column; ^d^ Identification by comparison of the mass spectral and retention index data; ^e^ QI, “quality index”, reflects the fit comparison of experimental mass spectrum and NIST library spectrum.

The antibacterial and antifungal activities of the fruit volatile oils of *A. cordifolia* and *C. subcordatum* showed promising antimicrobial activity (Table 3). *Alchornea cordifolia* oil demonstrated good antibacterial activity against the Gram-positive organism *S. aureus* (MIC = 78 μg/mL) and moderate antifungal activity against *A. niger* (MIC = 156 μg/mL); *C. subcordatum* oil exhibited moderate antibacterial activity against *B. cereus* and *S. aureus* (MIC = 156 μg/mL) and good antifungal activity (MIC = 39 μg/mL) against *A. niger*. It has been documented that Gram-positive bacteria are more sensitive to chemical compounds than Gram-negative bacteria due to differences in the structures of their cell walls; Gram-negative bacteria are less susceptible to hydrophobic small molecules such as essential oil components due to hydrophilic lipopolysaccharides in their outer membrane [41]. This is obvious by the sensitivity of the Gram-positive bacteria to the tested essential oils in the assay; however with differences in the degree of inhibition. The antimicrobial activities observed in the studied fruit essential oils can be attributed to the major constituents or a synergy between the major and some minor compounds. Consistent with the results of *A. cordifolia* essential oil, *Laportea aestuans* essential oil, rich in methyl salicylate (54.50%), has been shown to exhibit antimicrobial activity against *S. aureus*, *B. subtilis*, *P. aeruginosa*, *E. coli* and *C. albicans* at 200 mg/mL compared to the standard drug; however, it was more active against the fungi *Rhizopus stolon* and *A. niger* at 25 mg/mL [42]; *Pelargonium graveolens* essential oil, dominant in citronellol (26.7%), *inter alia*, and citronellol, also demonstrated strong inhibitory activity against *S. aureus* and *E. coli* [43,44]. Similarly, benzaldehyde (96.96% and 90.6%, respectively, in *P. myrtifolia* and apricot, *Prunus armeniaca*, seed essential oils) exhibited antimicrobial activity against *S. aureus*, *S. epidermidis*, *B. subtilis*, *P. aeruginosa*, *E. coli* and *C. albicans* [39,40]. Additionally, *Stachys cretica* essential oil (β-caryophyllene, 51.0%) and β-caryophyllene are reported to exhibit strong antimicrobial activity, particularly against *P. aeruginosa* and *B. subtilis* [45].

**Table 3:** Antimicrobial activity of *A. cordifolia* and *C. subcordatum* fruit volatile oils (MIC, μg/mL).

Sample	B.c	S.a	E.c	P.a	C.a	A.n
*A. cordifolia*	625	78	625	625	625	156
*C. subcordatum*	156	156	625	625	625	39
Positive control	1.22 ^a^	0.61 ^a^	2.44 ^a^	1.22 ^a^	0.61 ^b^	0.61 ^b^

B.c., *Bacillus cereus* (ATCC No. 14579); S.a, *Staphylococcus aureus* (ATCC No. 29213); E.c, *Escherichia coli* (ATCC No. 25922); P.a, *Pseudomonas aeroginosa* (ATCC No. 27853); C.a, *Candida albicans* (ATCC No. 10231); A.n, *Aspergillus niger* (ATCC No. 16401); ^a^ Gentamicin sulfate; ^b^ Amphotericin B; Negative control, DMSO had zero effect.

Both *A. cordifolia* and *C. subcordatum* fruit essential oils were screened for *in vitro* cytotoxic activity against Hs 578T human breast adenocarcinoma and PC-3 human prostatic carcinoma cells. Neither oil showed activity, however, with 0% kill at the concentrations tested.

## 4. Conclusions

The antimicrobial activities of *A. cordifolia* and *C. subcordatum* essential oils are a function of their distinct chemical profiles. The fruit essential oil of *A. cordifolia*, rich in methyl salicylate and citronellol, showed antibacterial activity against *S. aureus* and antifungal activity against *A. niger*. The antimicrobial activity can be attributed to these two major components, which have shown antimicrobial activities [46,47,48]. *C. subcordatum* fruit oil was active against *B. cereus*, *S. aureus*, and *A. niger*, but the activity is not likely due to the major component benzaldehyde, which is generally not antimicrobial [49], but rather a synergism between minor essential oil components. The promising antimicrobial activities of *A. cordifolia* and *C. subcordatum* essential oils are consistent with traditional uses of these medicinal plants.
